# Deregulated microRNAs in CD4^+^ T cells from individuals with latent tuberculosis *versus* active tuberculosis

**DOI:** 10.1111/jcmm.12205

**Published:** 2013-12-25

**Authors:** Yurong Fu, Zhengjun Yi, Jianhua Li, Ruifang Li

**Affiliations:** a Department of Medical Microbiology, Weifang Medical UniversityWeifang, China; b Department of Laboratory Medicine of Affiliated Hospital of Weifang Medical University, Key Laboratory of Clinical Laboratory Diagnostics in Universities of ShandongWeifang, China

**Keywords:** MicroRNA, CD4^+^ T cell, tuberculosis, latent infection, signalling pathway, molecular function

## Abstract

The mechanisms of latent tuberculosis (TB) infection remain elusive. Roles of microRNA (miRNA) have been highlighted in pathogen–host interactions recently. To identify miRNAs involved in the immune response to TB, expression profiles of miRNAs in CD4^+^ T cells from patients with latent TB, active TB and healthy controls were investigated by microarray assay and validated by RT-qPCR. Gene ontology (GO) and Kyoto Encyclopedia of Genes and Genomes (KEGG) pathway analysis were used to analyse the significant functions and involvement in signalling pathways of the differentially expressed miRNAs. To identify potential target genes for miR-29, interferon-γ (IFN-γ) mRNA expression was measured by RT-qPCR. Our results showed that 27 miRNAs were deregulated among the three groups. RT-qPCR results were generally consistent with the microarray data. We observed an inverse correlation between miR-29 level and IFN-γ mRNA expression in CD4^+^ T cells. GO and KEGG pathway analysis showed that the possible target genes of deregulated miRNAs were significantly enriched in mitogen-activated protein kinase signalling pathway, focal adhesion and extracellular matrix receptor interaction, which might be involved in the transition from latent to active TB. In all, for the first time, our study revealed that some miRNAs in CD4^+^ T cells were altered in latent and active TB. Function and pathway analysis highlighted the possible involvement of miRNA-deregulated mRNAs in TB. The study might help to improve understanding of the relationship between miRNAs in CD4^+^ T cells and TB, and laid an important foundation for further identification of the underlying mechanisms of latent TB infection and its reactivation.

## Introduction

Infection with *Mycobacterium tuberculosis* (*Mtb*) can result in active TB or, more commonly latent TB [1]. Overall, about 5–10% of persons with latent TB infection (LTBI) will go on to develop active TB at a later stage of their life [2,3]. About two billion persons worldwide are estimated to have LTBI, who represent an enormous reservoir of potential reactivation TB cases. Latency is associated with the state in which host is able to control infection, but unable to completely eradicate *Mtb* [4]. The exact underlying mechanisms of LTBI and its reactivation remain elusive. Further understanding of the mechanisms, by which *Mtb* establishes a latent state, eludes immune surveillance and responds to reactivation, is crucial for control of TB in the future.

MicroRNAs (miRNAs), endogenous non-coding RNAs (20–22 nucleotides), function *via* base-pairing with complementary sequences within mRNA molecules, usually resulting in gene silencing *via* translational repression or target degradation. Increasing evidence indicates that miRNAs play crucial roles in cell development, differentiation and communication [5,6]. Interestingly, some recent studies also provide compelling evidence that miRNAs are involved in the regulation of immune system, such as development and differentiation of B and T cells, antibody switching, as well as inflammatory mediators' release [7–9]. The potential clinical application of miRNAs as diagnostic or prognostic biomarkers has already been well demonstrated in many different types of cancer [10]. Mis-regulation of miRNAs could lead to great regulatory upheavals in the cell, perhaps resulting in cancer phenotype. However, compared to their well-known roles in cancer, roles of miRNAs in infectious disease, including those of bacterial origin, are still poorly understood.

Owing to the lack of a widely accepted animal model and cell model to study the pathogenesis of LTBI, population-based studies have been the best methods to reveal the complex pathogenic process of LTBI. A previous study used peripheral blood mononuclear cells (PBMCs) to elucidate the roles of miRNA in the transition from latent to active TB [11]. Cell-mediated immune responses are critical to overcome *Mtb* infection and CD4^+^ T cells are the main mediators of immune protection against *Mtb* infection [12]. To gain a better understanding of the underlying mechanism of LTBI and its reactivation, we investigated the miRNA expression profiles to test the hypothesis that miRNAs in CD4^+^ T cells might be involved in the transition from LTBI to active disease.

## Materials and methods

### Human subjects

Patients with active pulmonary TB were consecutively enrolled from the Affiliated Hospital of Weifang Medical University and Weifang Chest Hospital, China, from September 2011 to January 2012. The demographic and clinical characteristics of patients with active TB, LTBI and healthy controls were summarized in Table 1. Diagnosis of active pulmonary TB was based on typical clinical symptoms, such as cough, fever, pulmonary fibro-cavitation and infiltrates on chest radiograph, and at least one positive sputum smear. Biochemical tests and PCR method were used to identify *Mtb*. Patients were excluded if they had a history of diabetes, cancer or coinfection with other pathogens, such as HIV, HBV or HCV. Peripheral venous blood was drawn from the patients prior to initiation of anti-TB treatment. Individuals with LTBI and healthy control participants were recruited from the staff of two hospitals mentioned above, and participants were excluded if they had a prior history of TB, diabetes, cancer or other coinfected disease. Tuberculin skin test and interferon-γ (IFN-γ) release assay (IGRA; T-SPOT. TB, Oxford Immunuotec, Oxfordshire, UK) were used to distinguish LTBI participants from healthy controls. There were no significant differences among the groups in terms of age or gender.

**Table 1:** Characteristics of the participants

Characteristics	Control (*n* = 30)	LTBI (*n* = 28)	Active TB (*n* = 30)
Male/female	14/16	15/13	17/13
Age, mean (range) years	36.2 ± 13.4 (22–51)	38.7 ± 16.9 (23–53)	40.1 ± 19.8 (19–61)
TST test	Negative	Positive	Not applicable
IGRA	Negative	Positive	Not applicable
Diabetes	Negative	Negative	Negative
HIV	Negative	Negative	Negative
HBV	Negative	Negative	Negative
HCV	Negative	Negative	Negative
History of cancer	Negative	Negative	Negative

IGRA, Interferon-Gamma release assays; TST, Tuberculin skin test.

All patients with active pulmonary TB had clinical signs and symptoms; comprising, 83.3% cough, 66.8% weight loss, 65.6% fever, 60.2% night sweats and 43.4% hemoptysis. The healthy controls and cases with LTBI involved in the study were free of clinical symptoms of any infectious disease. The healthy controls, cases with LTBI and active TB patients were non-smokers.

The study was performed with the approval of Weifang Medical University local ethics committee and carried out in compliance with the Helsinki Declaration. All the participants provided informed consent before beginning the study.

### Isolation of CD4^+^ T cells

Peripheral venous blood was drawn from each participant and PBMCs were separated by density gradient centrifugation using Ficoll (TBD, China). Untouched CD4^+^ T cells were purified from PBMCs by negative selection using CD4^+^ T Cell Isolation Kit (R&D Systems, Minneapolis, MN, USA) according to the manufacturer's instructions. Flow cytometric analysis of a subset of samples showed that the purity of isolated CD4^+^ T cells was greater than 90%. Samples were stored in liquid nitrogen until RNA was extracted.

### RNA extraction and RNA quality control

Total RNA was isolated using Trizol reagent (Invitrogen, Carlsbad, CA,USA) and further purified with a miRNeasy mini kit (Qiagen, Dusseldorf, Germany) according to the manufacturer's protocol. RNA quality control procedure was followed to ensure that only high-quality RNA sample was used for microarray analysis. Spectrophotometry was performed to examine RNA purity and only sample with OD260/OD280 ∼2 and OD260/OD230 >1.8 was acceptable. Electrophoresis was used to analyse RNA integrity and only sample with 28S/18S >2 was adequate in the study.

### Detection of miRNAs expression and data analysis

For each sample (*n* = 4 for each group, randomly selected from the participants in Table 1), 1 μg total RNA was 3′-end-labelled with a miRCURYTM Hy3TM power labelling kit (Exiqon, Vedbaek, Denmark) and then subsequently hybridized to Exiqon Human miRCURY™ LNA Array (v.16.0) that contained ∼1223 capture probes (each probe being replicated four times on each array) according to the manufacturer's instructions. Arrays were scanned and each spot signal intensity was calculated by subtracting local background (based on the median signal intensity of the area surrounding each spot) from total intensity using Lowess Normalization (MIDAS, TIGR Microarray Data Analysis System). Intensity value of each miRNA was normalized to that of reference gene U6 RNA. After normalization, obtained average value for each miRNA was used for statistics, and a false discovery rate (FDR) value of less than 0.05 was considered statistically significant. The results were presented as fold change in miRNA expression. The expression profiles of the differentially expressed miRNAs between the groups were then subjected to hierarchical clustering using the Pearson correlation with average linkage to create a condition tree.

### RT-qPCR analysis

Five miRNAs were randomly selected from the deregulated miRNAs to be further confirmed using reverse transcription quantitative real-time PCR (RT-qPCR). RT reactions contained 700 ng RNA, 15 nM RT primers, 1× RT buffer, 0.25 mM each of dNTPs, 2 U/μl reverse transcriptase and 0.6 U/μl RNase inhibitor. The 20 μl reactions were incubated for 30 min. at 16°C, 42 min. at 42°C, and then 5 min. at 85°C. The cDNA product was used for the following PCR analysis. The 10 μl reactions included 1× Master Mix, 0.5 μM each primer and 2 μl cDNA. Reactions were incubated at 95°C for 10 min., followed by 40 cycles at 95°C for 10 sec. and 60°C for 1 min. on an ABI PRISM 7900 system (Applied Biosystems, Foster City, CA, USA). The primers (if needed, their sequences were available) were designed by optimization of parameters including melting temperature, GC content, complementarity and secondary structure, as well as primer length. At the same time, no template control (including all RT-PCR reagents except RNA template) was run to rule out cross-contamination of reagents and surfaces, and no amplification control (containing all RT-PCR reagents except reverse transcriptase) was used to eliminate genomic DNA contamination. All reactions were run in triplicate.

The Δ cycle threshold (Ct) values were obtained for each sample in each group for each miRNA tested. Human U6 small RNA level was used as the endogenous control. Normalized average value was used for statistics by the 2^−ΔΔCt^ method, and a *P* < 0.05 for each miRNA was considered statistically significant.

### Gene ontology and Kyoto encyclopedia of genes and genomes pathway analysis

Three target prediction databases (mirbase, miranda and targetscan) were used to identify potential target genes of the differentially expressed miRNAs. The overlapping targets predicted by the three databases were subjected to gene ontology (GO) analysis and Kyoto encyclopedia of genes and genomes (KEGG) pathway analysis. *P* < 0.05 was considered statistically significant.

### Measurement of IFN-γ mRNA expression by RT-qPCR

As T cells are reported to be the main producers of IFN-γ, which is critical for innate and adaptive immune response to *Mtb* infection, IFN-γ mRNA expression in CD4^+^ T cells was further assessed in the study. First-strand cDNA was generated using random primers. PCR was then performed with human IFN-γ specific primers (Forward 5′-3′ AGTTATATCTTGGCTTTTCA; Reverse 5′-3′ TTCGACTGATTAATAAGC). The data were analysed using the 2^−ΔΔCt^ method, and a *P* < 0.05 was considered statistically significant.

### Correlation between miR-29 expression and IFN-γ level in CD4^+^ T cells *in vitro*

CD4^+^ T cells freshly isolated from LTBI patient were seeded in triplicate in each well of 24-well plates at a concentration of 1 × 10^5^ cells/well in 500 μl culture medium containing serum (complete medium) at 37°C in a 5% CO_2_ incubator. Cells were then stimulated with purified protein derivative (PPD) from *Mtb* (25 μg/ml) for 24 hrs. RT-qPCR assay was used to measure miR-29 and IFN-γ mRNA expression. The data were analysed using the 2^−ΔΔCt^ method, and a *P* < 0.05 was considered statistically significant.

MiR-29 mimics (GenePharma, Shanghai, China) were used to further assess the effect of altered miR-29 level on IFN-γ production in stimulated CD4^+^ T cells with PPD *in vitro*. For the transfection procedure, CD4^+^ T cells were seeded in triplicate in each well of 24-well plates at a concentration of 1 × 10^5^ cells/well in 100 μl complete medium. Immediately, cells were either left untreated (medium only, untreated control) or transiently transfected with miR-29 mimics (70 nM) or negative mimics control (70 nM) using HiPerFect Transfection Reagent (Qiagen, Valencia, CA, USA) according to the manufacturer's protocols. Cells treated with transfection reagent but no mimics were used as mock control. Cells were incubated with transfection complexes under their normal growth conditions for 6 hrs. Then, 400 μl of complete medium containing 25 μg/ml PPD was added to each well and incubated for 24 hrs. RT-qPCR assay was used to determine IFN-γ mRNA expression. The data were analysed using the 2^−ΔΔCt^ method, and a *P* < 0.05 was considered statistically significant.

### Statistical analysis

Data were presented as mean ± SD. The differences in levels of miRNAs among groups were analysed using two-tailed *t*-tests or one-way anova for experiments with more than two groups. A *P* < 0.05 was considered statistically significant.

## Results

### Differential expression of miRNAs in CD4^+^ T cells from the different groups

**Figure 1:**
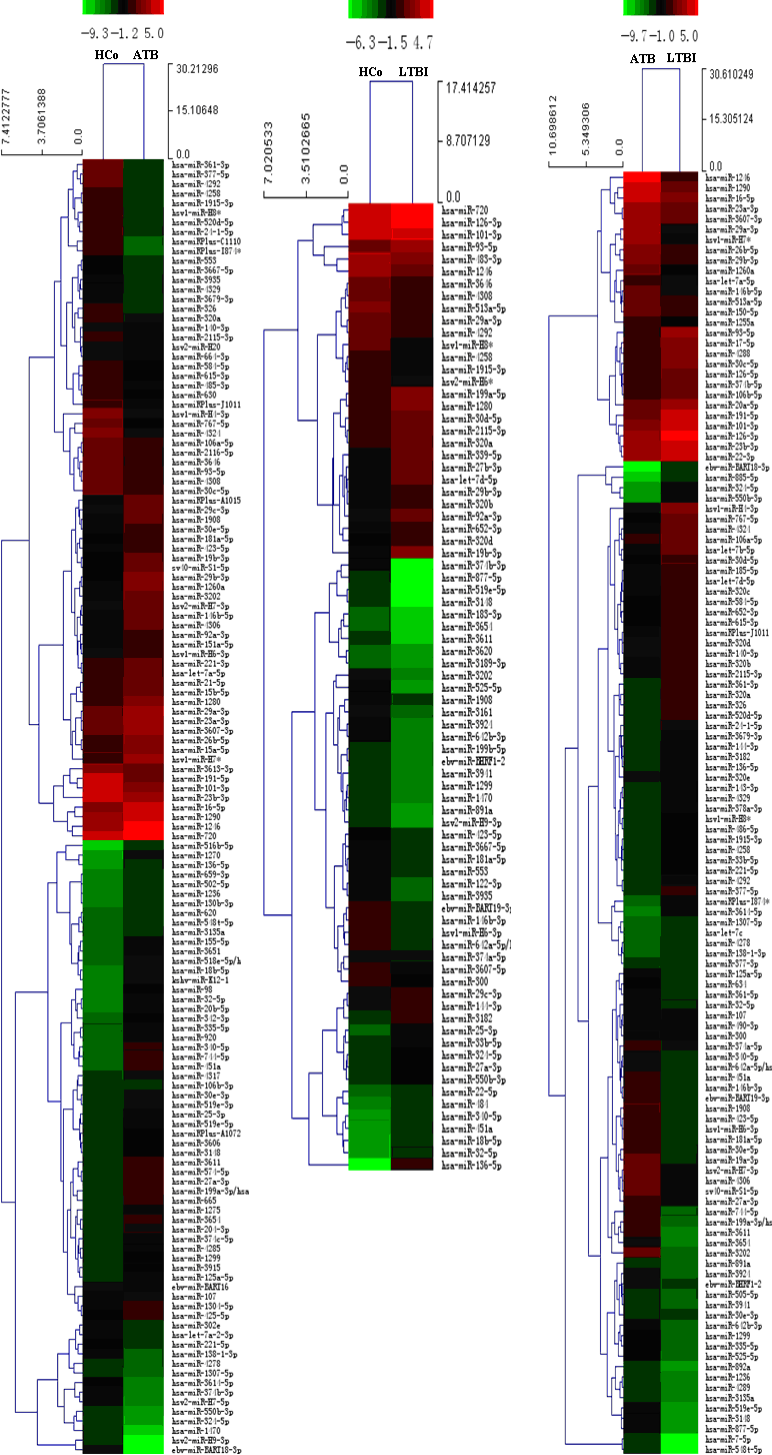
Differentially expressed miRNAs between each two groups. HCo: healthy control (*n* = 4); ATB: active TB (*n* = 4); LTBI: latent TB infection (*n* = 4). Red indicated high relative expression and green indicated low relative expression. MiRNA with expression fold change >2 and with FDR <0.05 was considered statistically significant.

To identify the most significant candidates, miRNAs with at least twofold expression change were selected. Under the criteria, 82 miRNAs were up-regulated and 53 miRNAs were down-regulated in the active TB group compared with the control group (Fig. 1); 33 miRNAs were increased and 46 miRNAs were decreased in the LTBI group compared with the control group (Fig. 1); 62 miRNAs were overexpressed and 62 miRNAs were downexpressed in the LTBI group compared with the active TB group (Fig. 1, Table 2; FDR < 0.05).

**Table 2:** Differently expressed miRNAs in the LTBI group *versus* the active TB group

Name	LTBI *versus* Active TB (up-regulated)	Name	LTBI *versus* Active TB (down-regulated)
hsa-miR-320c	2.04	hsa-miR-7-5p	0.01
hsa-miR-377-3p	2.07	hsa-miR-548t-5p	0.01
hsa-miR-4329	2.07	hsa-miR-3611	0.02
hsa-miR-652-3p	2.08	hsa-miR-3148	0.02
hsa-miR-143-3p	2.09	hsa-miR-3202	0.02
hsa-miR-106b-5p	2.14	hsa-miR-519e-5p	0.03
hsa-let-7d-5p	2.32	hsa-miR-199	0.03
hsa-miR-185-5p	2.32	hsa-miR-3654	0.03
hsa-miR-374b-5p	2.34	hsa-miR-877-5p	0.03
hsa-miR-20a-5p	2.35	hsa-miR-1246	0.05
hsa-miR-320e	2.36	hsa-miR-1908	0.06
hsa-miR-615-3p	2.37	hsa-miR-744-5p	0.08
hsa-miR-126-5p	2.39	hsa-miR-29a-3p	0.09
hsa-miR-4278	2.39	hsa-miR-525-5p	0.09
hsa-let-7c	2.41	hsa-miR-335-5p	0.09
hsa-miR-138-1-3p	2.46	hsa-miR-423-5p	0.09
hsa-miR-22-3p	2.52	hsa-miR-892a	0.10
hsa-miR-378a-3p	2.55	hsa-miR-19a-3p	0.10
hsa-miR-17-5p	2.69	hsv1-miR-H6-3p	0.11
hsa-miR-30c-5p	2.72	hsa-miR-181a-5p	0.11
hsa-miR-584-5p	2.74	hsv1-miR-H7^*^	0.11
hsa-let-7b-5p	2.95	hsa-miR-1299	0.13
hsa-miR-136-5p	2.99	hsa-miR-30e-5p	0.13
hsa-miR-23b-3p	3.07	ebv-miR-BART19-3p	0.14
hsa-miR-30d-5p	3.21	hsv2-miR-H7-3p	0.14
hsa-miRPlus-J1011	3.31	hsa-miR-3135a	0.15
hsa-miR-106a-5p	3.42	hsa-miR-4306	0.16
hsa-miR-1307-5p	3.53	hsa-miR-146b-3p	0.16
hsa-miR-486-5p	3.56	hsa-miR-1260a	0.16
hsa-miR-4288	3.65	hsa-miR-4289	0.16
hsa-miR-3182	3.66	hsa-miR-1290	0.17
hsv1-miR-H8^*^	3.66	hsa-miR-642b-3p	0.18
hsa-miR-144-3p	3.81	sv40-miR-S1-5p	0.18
hsa-miR-320d	4.02	hsa-miR-1236	0.19
hsa-miR-140-3p	4.03	hsa-miR-451a	0.20
hsa-miR-320b	4.03	hsa-miR-374a-5p	0.21
hsa-miR-1915-3p	4.19	hsa-miR-125a-5p	0.22
hsa-miR-4324	4.23	hsa-miR-27a-3p	0.23
hsa-miR-33b-5p	4.26	hsa-miR-340-5p	0.24
hsa-miR-4258	4.31	hsa-miR-3924	0.24
hsa-miR-24-1-5p	4.38	hsa-miR-26b-5p	0.24
hsa-miR-126-3p	4.53	hsa-miR-642	0.25
hsa-miR-3679-3p	4.62	hsa-miR-3941	0.27
hsa-miR-101-3p	4.71	hsa-miR-891a	0.30
hsa-miR-191-5p	4.74	hsa-miR-3607-3p	0.31
hsa-miR-221-5p	5.04	ebv-miR-BHRF1-2	0.31
hsa-miR-2115-3p	5.53	hsa-miR-505-5p	0.32
hsa-miR-767-5p	5.82	hsa-miR-23a-3p	0.34
hsa-miR-520d-5p	6.57	hsa-miR-146b-5p	0.36
hsa-miR-93-5p	6.63	hsa-miR-32-5p	0.37
hsa-miR-320a	7.29	hsa-miR-150-5p	0.40
hsa-miR-4292	7.43	hsa-miR-29b-3p	0.40
hsa-miR-377-5p	8.32	hsa-miR-634	0.41
hsa-miR-3614-5p	9.60	hsa-miR-107	0.41
hsa-miR-326	10.1	hsa-miR-16-5p	0.41
hsa-miR-361-3p	13.0	hsa-miR-361-5p	0.42
hsa-miRPlus-I874	13.6	hsa-let-7a-5p	0.43
hsv1-miR-H4-3p	19.9	hsa-miR-513a-5p	0.44
hsa-miR-885-5p	21.0	hsa-miR-1255a	0.44
hsa-miR-550b-3p	21.1	hsa-miR-490-3p	0.45
hsa-miR-324-5p	34.3	hsa-miR-30e-3p	0.47
ebv-miR-BART18-3p	120	hsa-miR-300	0.47

After normalization, obtained average values for each miRNA spot were used for statistics. MiRNA with expression fold change >2 and with FDR <0.05 was considered statistically significant.

**Figure 2:**
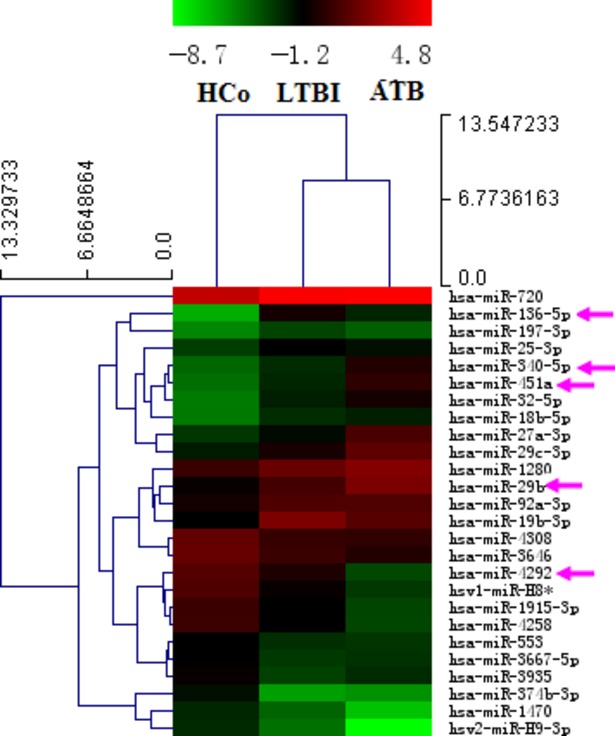
Differentially expressed miRNAs among the three groups. HCo: healthy control (*n* = 4); ATB: active TB (*n* = 4); LTBI: latent TB infection (*n* = 4). Red indicated high relative expression and green indicated low relative expression. MiRNA with expression fold change >2 and with FDR <0.05 was considered statistically significant. MiRNAs marked with arrow were randomly selected for further confirmation by RT-qPCR.

A total of 27 miRNAs (Fig. 2) were differentially expressed among the three groups (FDR < 0.05). Among the 27 miRNAs, 26 miRNAs had similar expression trend in both the active TB and LTBI group compared with the control group; 14 miRNAs were increased; and 12 miRNAs were decreased in both the active TB and LTBI group. Among the 27 miRNAs, 11 miRNAs (miR-136-5p, miR-340-5p, miR-451a, miR-32-5p, miR-27a-3p, miR-29a, miR-29b, miR-4292, miR-H8*, miR-1915-3p, miR-4258) were differentially expressed between the active TB group and the LTBI group; five miRNAs (miR-136-5p, miR-4292, miR-H8*, miR-1915-3p, miR-4258) were up-regulated in the LTBI group; and six miRNAs were down-regulated. Among the 11 miRNAs differentially expressed between the active TB group and the LTBI group mentioned above, six miRNAs (miR-136-5p, miR-340-5p, miR-451a, miR-32-5p, miR-27a-3p, miR-29b) were increased and four miRNAs (miR-4292, miR-H8*, miR-1915-3p, miR-4258) were decreased in both the LTBI and active TB group compared with the control group.

### Validation of microarray results by RT-qPCR

**Figure 3:**
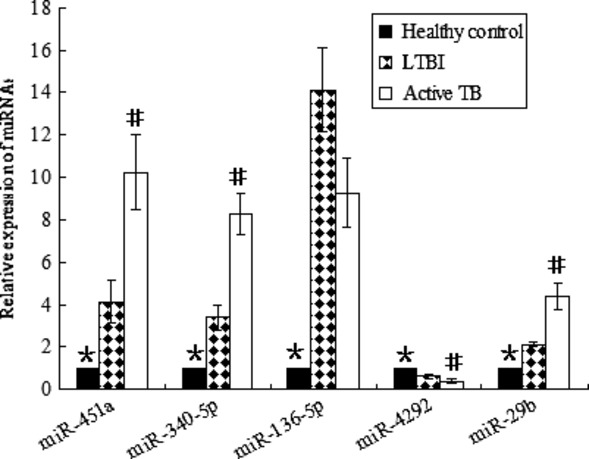
Confirmation of miRNA level by RT-qPCR. Relative expression of each miRNA was normalized to U6 level. MiR-451a, miR-340-5p and miR-29b were obviously increased, whereas miR-4292 was significantly decreased in the active TB group compared with the LTBI group. MiR-136-5p showed no difference between the active TB group and the LTBI group. All of them except miR-4292 were significantly overexpressed in both the LTBI and the active TB group compared with the healthy controls. *Significant difference between the healthy controls (*n* = 30) and the LTBI group (*n* = 28), as well as between the healthy controls and the active TB group (*n* = 30). #Significant difference between the LTBI group and the active TB group. *P* < 0.05 was considered statistically significant. Error bars in graphs referred to standard deviation. Each reaction was run three separate times, with technical triplicates in each reaction.

To confirm the microarray results, five miRNAs (miR-451a, miR-340-5p, miR-136-5p, miR-4292, miR-29b) were randomly selected for further confirmation using RT-qPCR. Among the five selected miRNAs, all of them except miR-136-5p were differentially expressed between the active TB group and the LTBI group (*P* < 0.05); three miRNAs (miR-451a, miR-340-5p, miR-29b) were increased in the active TB group; and only miR-4292 was decreased (Fig. 3). Among the five selected miRNAs, four miRNAs (miR-451a, miR-340-5p, miR-136-5p, miR-29b) were down-regulated in comparison of the control group with the LTBI group, as well as the control group with the active TB group (*P* < 0.05). The results were generally consistent with the microarray data.

### GO and KEGG pathway analysis of the deregulated miRNAs

**Figure 4:**
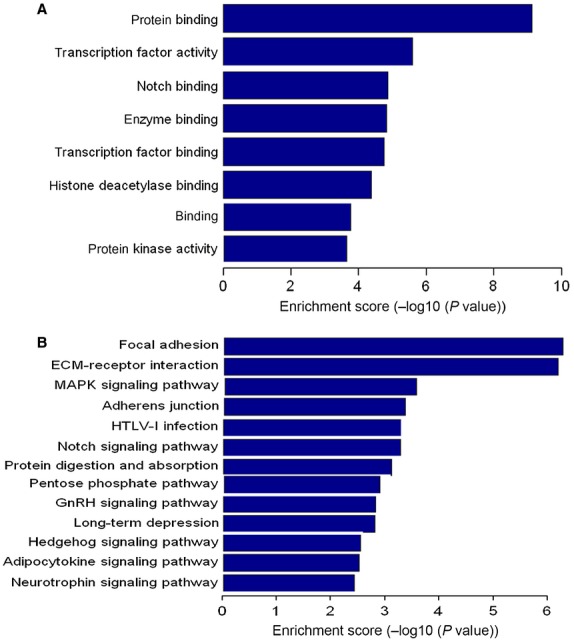
GO and KEGG pathway analysis of the deregulated miRNAs. Enrichment score is equal to −log10 (*P*-value). The higher the enrichment score is, the more specific the corresponding function is. (A) significant GOs. (B) significant signalling pathways. KEGG: Kyoto Encyclopedia of Genes and Genomes; GO: Gene ontology. BH-corrected *P* < 0.05 was considered statistically significant.

As the number of experimentally validated miRNA targets is very limited, to explore the functions of 27 differentially expressed miRNAs among the three groups, we predicted their potential target genes. The biology of the predicted target genes was further explored regarding their molecular functions and involvement in known regulatory pathways. The predicated miRNA targets were categorized into several biological functions, of which protein binding and focal adhesion were shown to be the most enriched ones (Fig. 4A and B; BH-corrected *P* < 0.05). Among the enriched signalling pathways, only a few ones, such as mitogen-activated protein kinase (MAPK) signalling pathway, have been found to be involved in response to TB infection [13,14]; however, other signalling pathways have never been reported, such as extracellular matrix (ECM)–receptor interaction and GnRH signalling pathway.

### Measurement of IFN-γ mRNA expression

Both microarray and RT-qPCR results showed that miR-29 levels in CD4^+^ T cells were decreased in comparisons of the control group with the LTBI group, as well as the control group with the active TB group. On the basis of IFN-γ as one target of miR-29 [15], it has been suggested that up-regulated miR-29 in CD4^+^ T cells might in turn inhibit IFN-γ mRNA expression in both the LTBI and active TB group. Consistent with our hypothesis, IFN-γ mRNA levels were decreased in both the LTBI group and the active TB group compared with the controls, and reduced more in the active TB group compared with the LTBI group (*P* < 0.05). The results indicated an inverse correlation between miR-29 level and IFN-γ mRNA expression in CD4^+^ T cells response to *Mtb* infection.

### MiR-29 mimics down-regulated IFN-γ production *in vitro*

**Figure 5:**
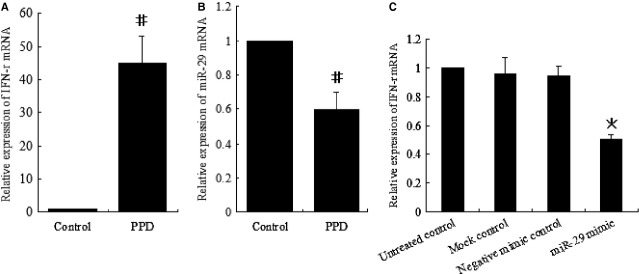
Correlation between miR-29 expression and interferon-γ (IFN-γ) level in CD4^+^ T cells *in vitro*. (A and B) RT-qPCR analysis of IFN-γ (A) and miR-29 mRNA (B) in CD4^+^ T cells left unstimulated (control) or stimulated with PPD for 24 hrs. (C) RT-qPCR analysis of IFN-γ mRNA in CD4^+^ T cells transfected with miR-29 mimics or control mimics. #Significant difference between the control and PPD. *Significant difference between miR-29 mimics and the controls. *P* < 0.05 was considered statistically significant. Error bars in graphs referred to standard deviation. All experiments were repeated at least three times.

To further determine the correlation between the expression of miR-29 and that of IFN-γ in CD4^+^ T cells response to TB infection, several experiments were performed. We found that activated CD4^+^ T cells with PPD had higher expression of IFN-γ mRNA, but lower miR-29 mRNA expression than did non-activated CD4^+^ T cells (Fig. 5A and B), which demonstrated an inverse correlation between the expression of miR-29 and that of IFN-γ in activated CD4^+^ T cells with PPD.

MiR-29 mimics (synthetic miRNAs that mimic the function of endogenous miR-29) were used to further assess the effect of increased miR-29 level on IFN-γ production in activated CD4^+^ T cells with PPD. We found that miR-29 mimics significantly inhibited IFN-γ production in activated CD4^+^ T cells with PPD (Fig. 5C), which suggested that miR-29 might be involved in negative regulation of IFN-γ in response to TB infection.

## Discussion

Many individuals who have LTBI never develop TB disease, but some individuals who have LTBI are more likely to develop TB disease than others at some point during their lifetime. The detailed mechanisms still remain largely unknown. The immune response to TB infection is complex and multiple host factors are involved in this process [16]. A recent study reported that 17 miRNAs were differentially expressed in PBMCs from participants with LTBI, active TB and healthy controls [11]. It is known that CD4^+^ T cells and their derived cytokines are crucial for protection against *Mtb* [17]. Considering the important roles of CD4^+^ T cells in the immune response to TB infection, we focused on identification of the deregulated miRNAs in CD4^+^ T cells related to TB infection in the study. CD4^+^ T cells are an important subset of PBMCs; hence, it has been suggested that the deregulated miRNA profiles in CD4^+^ T cells might be largely consistent with those in PBMCs during TB infection. In this study, it was worth mentioning that only deregulated miR-451 in PBMCs [11] was also differentially expressed in CD4^+^ T cells, whereas other 16 differentially expressed miRNAs in PBMCs [11] were not deregulated in CD4^+^ T cells. These data indicated that the major cell type in PBMCs that showed 17 deregulated miRNAs [11] might not be CD4^+^ T cells, and suggested that response of PBMCs to TB infection might not be consistent with that of CD4^+^ T cells to TB infection. Further studies are needed to explore the inconsistency between our results and published data [11].

In this study, miR-136-5p was the most overexpressed and miR-374b-3p was the most underexpressed miRNAs in the LTBI group compared with the healthy controls respectively; miR-1270 was the most increased and miR-BART18-3p was the most decreased miRNAs in the active TB group compared with the healthy controls respectively; miR-324-5p was the most up-regulated and miR-548t-5p was the most down-regulated miRNAs in the LTBI group compared with the active TB group respectively. Many deregulated miRNAs in the study, such as let-7b [18], miR-320 [19] and miR-144 [20], have been discovered to be involved in specific signalling pathways. However, the biological functions of most differentially expressed miRNAs in CD4^+^ T cells, such as miR-548t-5p and miR-BART18-3p, are still largely unknown.

Twenty-seven miRNAs were found to be differentially expressed among the three groups. Interestingly, the majority of the deregulated miRNAs had similar expression trend in both the active TB and the LTBI group compared with the healthy controls, which indicated that both active TB and LTBI might share, at least partly, similar regulatory mechanisms. To identify miRNAs in CD4^+^ T cells that might be correlated with resistance or susceptibility to TB infection, we focused on the differentially expressed miRNAs between the LTBI group and the active TB group. Among the deregulated 27 miRNAs among the three groups, five miRNAs were increased, whereas six miRNAs were decreased in the LTBI group compared with the active TB group. The functions of miR-4292, miR-H8*, miR-1915-3p and miR-4258 remain unknown. A few miRNAs were found to be associated with carcinogenesis in various organs. MiR-136 was found to promote glioma cell apoptosis by targeting AEG-1 and Bcl-2 [21]. MiR-340 could inhibit breast cancer cell migration and invasion through targeting oncoprotein c-Met [22]. MiR-32 was shown to be involved in castration-resistant prostate cancer [23]. Our results suggested an additional mode of actions for these deregulated miRNAs. Several miRNAs were well known to regulate various immune systems. MiR-451 could regulate dendritic cell cytokine to response to influenza infection [24], and appeared as a very promising candidate for miRNAs involved in response to pathogen infection [25]. MiR-27a-3p was found to promote cell proliferation in glioma cells *via* cooperative regulation of MXI1 [26]. Another study showed that TGF-β–associated miR-27a inhibited dendritic cell–mediated differentiation of Th1 and Th17 cells by targeting [TGF (transforming growth factor) beta activated kinase 1 binding protein (TAB 3)], p38 MAPK, MAP2K4 and MAP2K7 [27]. Our results suggested that these miRNAs might also be involved in alterations of CD4^+^ T cells response to *Mtb* infection. It was worth noting that miR-29a, miR-29b and miR-29c were overexpressed in the active TB group compared with the healthy controls; miR-29b and miR-29c were up-regulated, while miR-29a was down-regulated in the LTBI group compared with the healthy controls. Antoher study showed that miR-29a was specifically increased after mycobacterial infection of human macrophages [28], which was well consistent with our recent data that miR-29a was increased by 5.21-fold in sputum and increased by 11.9-fold in serum in the active TB group compared with the healthy controls respectively [29]. These results suggested that miR-29 family members were involved in innate and adaptive immune responses to *Mtb* infection. *Mtb* infection can be controlled by effective activation of CD4^+^ T lymphocytes. Activated CD4^+^ T cells can secrete Th1 cytokines such as IFN-γ, which has a critical role in immune response to TB infection [30]. Considering the role of miR-29 in the suppression of protective immune response to intracellular pathogens by down-regulation of IFN-γ, it has been suggested that IFN-γ mRNA expression in CD4^+^ T cells might be decreased in the active TB group and the LTBI group compared with the healthy controls. Consistent with our hypothesis, our data showed that IFN-γ mRNA levels were down-regulated in both the LTBI group and the active TB group compared with the healthy controls, and IFN-γ mRNA expression was decreased more in the active TB group compared with the LTBI group, which could partly explain one mechanism that overexpressed miR-29 in the active TB group might inhibit CD4^+^ T cells response to TB infection by suppression of IFN-γ–mediated signalling pathway. MiR-29 was up-regulated, whereas IFN-γ was decreased in the active TB group compared with the LTBI group. miR-29 mimics obviously suppressed IFN-γ mRNA production in activated CD4^+^ T cells with PPD *in vitro*. These data suggested that miR-29 in CD4^+^ T cells might act as a negative regulator of immune response against TB infection. It was unknown whether miR-29 was involved in the transition from LTBI to actvie TB by inhibiting IFN-γ. However, one thing we can be sure of was that miRNAs might play important roles in the pathogenesis of TB infection by regulation of related genes expression and miR-29 in CD4^+^ T cells might disturb the delicate balance for control of *Mtb* infection. Further experiments were needed to test the effect of modulating miR-29 level on IFN-γ production during active TB infection and latent TB infection *in vivo*.

Based on the reported and predicted target genes of these deregulated miRNAs, KEGG pathway and GO analysis were used to identify significantly enriched functions and signalling pathways associated with TB infection. Our data showed that MAPK, focal adhesion and ECM–receptor interaction signalling pathways were abundant among the significantly enriched ones. Furthermore, protein binding, transcription factor and notch binding represented the significantly enriched GOs. These findings suggested that various cellular processes and signalling pathways were involved in the TB infection. To narrow the scope of study and evaluate the most significant candidates, miRNAs and their possible target genes, which were in the intersection of protein binding and MAPK signalling pathway, might be the focus of the future studies.

In summary, we identified specific miRNAs that were differentially expressed among the active TB group, the latent TB group and the healthy controls, which might potentially be used to discriminate active TB from latent TB infection. These findings opened up a new and interesting avenue towards an improved understanding of the relationship between miRNAs homeostasis in CD4^+^ T cells and TB infection. However, the relationship between TB infection and miRNAs in CD4^+^ T cells was just beginning to be explored and it was difficult to synthesize our current results to reach a definitive conclusion based on this single study. Further research is needed to determine the roles of miRNAs in the conversion of LTBI to active disease.
